# White matter hyperintensities in cholinergic pathways may predict poorer responsiveness to acetylcholinesterase inhibitor treatment for Alzheimer’s disease

**DOI:** 10.1371/journal.pone.0283790

**Published:** 2023-03-31

**Authors:** Li-Hua Lee, Shu-Ching Wu, Cheng-Feng Ho, Wan-Lin Liang, Yi-Chien Liu, Chia-Ju Chou

**Affiliations:** 1 Department of Neurology, Cardinal Tien Hospital, New Taipei City, Taipei, Taiwan; 2 Department of Radiology, Cardinal Tien Hospital, New Taipei City, Taipei, Taiwan; 3 Department of Medical Research, Far Eastern Hospital, New Taipei City, Taipei, Taiwan; 4 Department of Education and Research, Medical school of Fu-Jen University, New Taipei City, Taipei, Taiwan; 5 Geriatric Behavioral Neurology Project, Tohoku University New Industry Hatchery Center (NICHe), Sendai, Japan; The Hong Kong Polytechnic University, HONG KONG

## Abstract

**Background:**

Acetylcholinesterase inhibitor (AChEI) drug regimens are the mainstay treatment options for patients with Alzheimer’s disease (AD). Herein, We examined the association between clinical response to AChEI and white matter hyperintensities on magnetic resonance imaging (MRI) scan at baseline.

**Methods:**

Between 2020 and 2021, we recruited 101 individuals with a clinical diagnosis of probable AD. Each participant underwent complete neuropsychological testing and 3T (Telsa) brain magnetic resonance imaging. Responsiveness to AChEI, as assessed after 12 months, was designated as less than two points of regression in Mini-Mental State Examination scores (MMSE) and stable clinical dementia rating scale. We also evaluated MRI images by examining scores on the Cholinergic Pathways Hyperintensities Scale (CHIPS), Fazekas scale, and medial temporal atrophy (MTA) scale.

**Results:**

In our cohort, 52 patients (51.4%) were classified as responders. We observed significantly higher CHIPS scores in the nonresponder group (21.1 ± 12.9 vs. 14.9 ± 9.2, P = 0.007). Age at baseline, education level, sex, Clinical Dementia Rating sum of boxes scores, and three neuroimaging parameters were tested in regression models. Only CHIPS scores predicted clinical response to AChEI treatment.

**Conclusion:**

WMHs in the cholinergic pathways, not diffuse white matter lesions or hippocampal atrophy, correlated with poorer responsiveness to AChEI treatment. Therefore, further investigation into the role of the cholinergic pathway in AD is warranted.

## Introduction

Alzheimer’s disease (AD) is the most common neurodegenerative disease affecting older adults. Although various chronic conditions, genetic and environmental factors contribute to AD, the exact causes of AD development and progression remain unclear. A notable postulation in AD research is that cognitive decline and behavioral symptoms are linked to reductions in acetylcholine (ACh) in the brain. This neurotransmitter is involved in learning, memory, attentional processes, circadian rhythmicity [1, 2]. A systematic review of 22 clinical trials reported that patients with AD exhibited improvements in cognitive function following acetylcholinesterase inhibitor (AChEI) treatment [3]. A meta-analysis of studies on AChEI treatment for AD noted the mitigation of neuropsychiatric symptoms and improvements in the ability of performing activities of daily living [4]. Thus, restoring cholinergic activity in the brain constitutes a mainstream treatment for AD.

Responsiveness to AChEIs varies among individuals, and many factors relate to it. In one recently published article, Pozzi et al. [5] reviewed predictors of responsiveness AChEIs according to many categories, such as neuroimaging predictors, factors related to drugs (plasma drugs concentration, doses of AChEIs, concomitant drug use), factors related to drug metabolism and genetic polymorphism, the underlying cognitive impairment, demographic characteristics, presence of underlying comorbidities, etc. In his systematic review, the authors reported that response to AChEIs has somehow correlated with certain features of cholinergic deficits (hallucination, fluctuating cognition, substantia innominate atrophy) and preserved cholinergic neurons, which were detected by EEG studies and brain perfusion studies [5]. In addition, pathological risk factors related to AD may correlate with treatment response. For example, apolipoprotein E (APOE)-e4 allele status, by far the most relevant genetic risk factor for AD, has been linked with both responsiveness and nonresponsiveness to AChEIs in many studies although a meta-analysis [6] indicated that APOE4 does not significantly affect responses to AChEI treatment.

Hippocampal atrophy and white matter hyperintensities (WMHs), the most notable MRI findings of AD, are highly correlated with disease progression and correspondent with underlying pathological changes in earlier studies [7, 8]. According to the cholinergic hypothesis, there was a selective loss of cholinergic neurons in AD [9]. Nowadays, many researchers used advanced neuroimaging methods to detect microstructural changes in these cholinergic tracts and neurons as biomarkers of AD patients. Several studies proved that either cholinergic tract damage or cholinergic neuron degeneration at the microstructural level correlated with cognitive impairment in AD [10, 11]. A recent study also reported that the integrity of cholinergic white matter pathways was reduced in all stages of AD continuum including individuals with subjective cognitive decline [12].

These neuroimaging parameters have been employed in predicting responses to AchEIs in many studies with inconsistent results [5]. Some studies have indicated that hippocampal atrophy is a stronger determinant of responses to AChEI treatment [13, 14] whereas others have concluded that WMHs are the stronger determinant [15, 16]. There were very few studies that used cholinergic pathway hyperintensities scales to predict the response of AChEIs [17, 18]. An overview of findings from relevant studies, including significant neuroimaging parameters, is presented in Table 1.

**Table 1:** Previous studies of correlation of neuroanatomical markers and response of cholinesterase inhibitors in Alzheimer’s disease.

Authors	Sample size	Drug	Follow-up Duration	Neuroanatomical parameters	Significant parameters
Teipel et al. [19]	124	Not identified	1 year	Basal forebrain volume, hippocampal volume	Basal forebrain volume
Wu et al. [15]	196	D	1 year	ARWMC	WMC over BG and frontal lobe
Teipel et al. [13]	215	D	1 year	Basal forebrain volume, hippocampal volume	Hippocampal volume
Gallucci et al. [20]	70	Not identified	1 year	Localized cortical atrophy	None
Cheng et al. [14]	353	D, R, M, G	46 months	MTA, WMHs	MTA
Fukui et al. [21]	551	D, R, M, G	1 year	PVHs	PVHs
Devine et al. [16]	243	D, R	9 months	WMHs	WMHs
Fukui et al. [22]	50	D	1 year	PVHs	PVHs
Connelly et al. [23]	160	Not identified	6 months	WMLs	WMLs
Blasko et al. [24]	20	D	18 months	Subcortical vascular lesions	None
Amar et al. [25]	72	Tacrine	3 months	Leukoaraiosis	None
Fukui et al. [18]	67	D	2 years	WMHs, CHIPS	WMHs, CHIPS
Hongo et al. [17]	41	D	24 months	CHIPS, rCBF	rCBF
Ho et al. [26]	87	R	1 year	Periventricular and deep WMH	Hypertension

Note: Not identified means that the authors did not identify what type of cholinesterase inhibitors were used in the study. Significant parameters refer to neuroanatomical parameters significantly associated with AChEI responses.

D donepezil; R rivastigmine; M memantine; G galantamine; WM white matter; WMC WM changes; ARWMC age-related WMC; MTA medial temporal atrophy; WMH WM hyperintensity; PVH periventricular hyperintensity; CHIPS Cholinergic Hyperintensities Pathway Scale; WML WM lesion; BG basal ganglia

The responses to AChEI treatment can also correlate with the integrity of the cholinergic system in the brain. Neurodegeneration can compromise this integrity through cortical-Ach-secreting neurons or cholinergic pathways damage [27]. The intactness of cortical-ACh-secreting neurons also comes from intact cortical cholinergic activities and basal forebrain neurons. A study measuring cortical acetylcholinesterase activity through positron emission tomography (PET) observed reduced cortical cholinergic activity, which is correlated with poor responses to AChEIs in patients with AD [28]. In a longitudinal study involving patients with AD undergoing AChEI treatment, the reduction in the basal forebrain volume was a significant predictor of the global cognitive response [19]. In sum, this evidence demonstrates the importance of intact cortical-Ach-secreting neurons to AChEI responsiveness.

Cholinergic pathways, comprising bundles of AChE-rich fiber projecting from basal forebrain neurons, constitute another essential factor influencing cholinergic integrity. Cholinergic pathways in postmortem human brains were examined through the acetylcholinesterase histochemistry method [29]. The current study used the Cholinergic Pathways Hyperintensities Scale (CHIPS), a visual rating system, to assess lesions on cholinergic projections [30]. We postulated that WMHs in the cholinergic pathways would be a determinant of AChEI responsiveness. Individuals with higher CHIPS scores may have less intact cholinergic pathways and poorer responsiveness to AChEIs.

## Method

### Study design

We performed a retrospective cohort study between 2020 and 2021, recruiting participants with clinical diagnosis of probable AD from the memory clinic of Cardinal Tien Hospital. To classify responses to AChEI treatment, we reviewed and analyzed patients’ baseline data (demographic information, clinical characteristics, and neuroimaging data from medical records) and cognitive changes over time, as evaluated from their Mini-Mental State Examination (MMSE), Clinical Dementia Rating (CDR), and CDR sum of boxes (CDR-SB) scores. We obtained the informed consent from all participants and/or their legal guardian(s). The study was conducted in accordance with the tenets of the Declaration of Helsinki, and the study protocol was approved by the Institutional Review Board (IRB) of Cardinal Tien Hospital (IRB no.: CTH-107-2-1-067).

### Participant recruitment

We recruited individuals with probable AD (from mild cognitive impairment to moderate dementia) and all participants had completed work-up for dementia survey at their initial visit and had already ruled out possible causes of reversible dementia. They all met the clinical diagnosis of probable Alzheimer’s disease (AD) dementia according to National Institute on Aging and the Alzheimer’s Association 2011 criteria (NIA-AA 2011) [31, 32]. All these patients were persistently taking cholinesterase inhibitor; donepezil, starting from 5 mg and slowly titrated up to 10 mg for at least 12 months. We excluded patients with parkinsonism, dementia caused by other neurodegenerative disorders, vascular dementia, and mixed-type dementia. The term vascular dementia means that the onset of cognitive deficits is temporally related to one or more cerebrovascular events as supported by history taking, focal neurological deficits, and neuroimaging evidence [33], and mixed type dementia means co-existence of typical AD-like dementia with a history of cerebrovascular accident previously [34]. Those who lost to follow up and didn’t have their initial brain MRI before taking Donepezil were also excluded.

Before treatment, all these patients underwent neuropsychological assessments by using Chinese version of CDR, and Mini-Mental State Examination (MMSE) and we collected these data as baseline cognitive functions. Their age, MMSE scores, and global CDR scores ranged from 70 to 85 years, 14 and 24 points, and 0.5 and 2 points, respectively [Table 2].

**Table 2:** Baseline demographic, neuroimaging, and cognitive functions of individuals with probable Alzheimer’s disease.

	Responders (n = 52)	Non-responders (n = 49)	P value
Age at baseline, years (mean) (SD)	76.6 (7.4)	79.3 (7.1)	0.059
Education, years (mean) (SD)	7.7	7.8	0.900
Women, n (%)	34 (65.4)	30 (61.2)	0.665
Hypertension, n (%)	35 (67.3)	33 (67.3)	0.99
Type 2 diabetes mellitus, n (%)	21 (40.4)	16 (32.7)	0.42
Baseline MMSE score (mean) (SD)	19.0 (5.1)	19.7 (5.1)	0.503
Baseline CDR score (mean) (SD)	0.75 (0.4)	0.83(0.5)	0.362
CDR 0.5, n (%)	34 (65.4)	30 (61.2)	0.567
CDR 1, n (%)	14 (26.9)	12 (24.5)	0.567
CDR 2, n (%)	4 (7.7)	7 (14.3)	0.567
Baseline CDR-SB score (mean) (SD)	4.2 (2.8)	4.6 (3.5)	0.527
Total MTA score, mean (SD)	2.2 (2.5)	2.1 (1.9)	0.879
CHIPS score, mean (SD)	14.9 (9.2)	21.1 (12.9)	0.007*
Fazekas score, mean (SD)	1.2 (0.6)	1.4 (0.6)	0.136

Note: SD standard deviation; MMSE Mini-Mental State Examination; CDR Clinical Dementia Rating; CDR-SB CDR sum of boxes; MTA medial temporal atrophy; CHIPS Cholinergic Hyperintensities Pathway Scale

* *p<0*.*05*. Hypertension means blood pressure >140/90 mmHg during OPD visit for more than two times 24 hours apart or if taking anti-hypertensive drugs and type 2 diabetes mellitus was use diagnosed according to criteria of American Diabetes Association (29).

And we regarded time as T0; before treatment and as T1; after one year of treatment. All patients had regular follow-up visit and MMSE and CDR were reassessed annually and determined the responsiveness of treatment. We defined responsiveness as “less than expected cognitive decline”, in agreement with part of the literature on AChEI response predictors” [26]. In one longitudinal follow-up study for probable AD patients, Clark CM et al, found that the clinically meaningful annual MMSE decline was greater than 3 [35]. Thus we defined good responsiveness of the acetylcholinesterase inhibitor only when the participants fulfilled the following two criteria; (1) follow-up MMSE score at T1 must not be regressed more than 2 from the baseline T0 (2) there must not be the regression of CDR score at T1 as compared to those at T0. Then, participants were divided into responder and non-responder groups according to the above criteria.

#### Image acquisition

All participants received whole-brain MRI scan (3.0 T, MAGNETOM Skyra, Siemens, Taipei, Taiwan). The MRI acquisition protocol is presented as follows: whole-brain axial and sagittal T2-weighted fluid-attenuated inversion recovery (FLAIR) sequence (FLAIR repetition time [TR]/time to echo [TE] = 3550/98 ms) with a 5-mm slice thickness, axial T1-weighted sequence (TR/TE = 2200/3 ms) with a 3-mm slice thickness, and high-resolution coronal T1-weighted three-dimensional (3D) magnetization-prepared rapid gradient-echo image (TR/TE = 2200/5 ms) with a 1-mm slice thickness. We used axial view of T2-weighted-Fluid-Attenuated Inversion Recovery (FLAIR) sequence for rating Fazekas scale and CHIPS and high resolution T1 weighted 3D images for rating MTA scale. Two well-trained raters (YCL and LHL) who were blinded to the clinical data assessed all the MRI images.

#### Medial temporal atrophy scale

The medial temporal atrophy (MTA) scale is a visual rating system for assessing the size of the hippocampus relative to the surrounding cerebrospinal fluid space [36]. For each side of the hippocampus, the scale ranges from 0 to 4 points. A coronal T1-weighted 3D brain MRI sequence was employed. We measured both sides of the hippocampus and calculated the total MTA score by summing the scores for the left and right sides. The intra-class correlation coefficient was 0.91.

#### Fazekas scale

Scores on the Fazekas scale, which is used to quantify the degree of diffuse WMHs [37], ranges from 0 (normal) to 3 (confluent). The intra-class correlation coefficient was 0.92.

#### CHIPS

**Fig 1:**
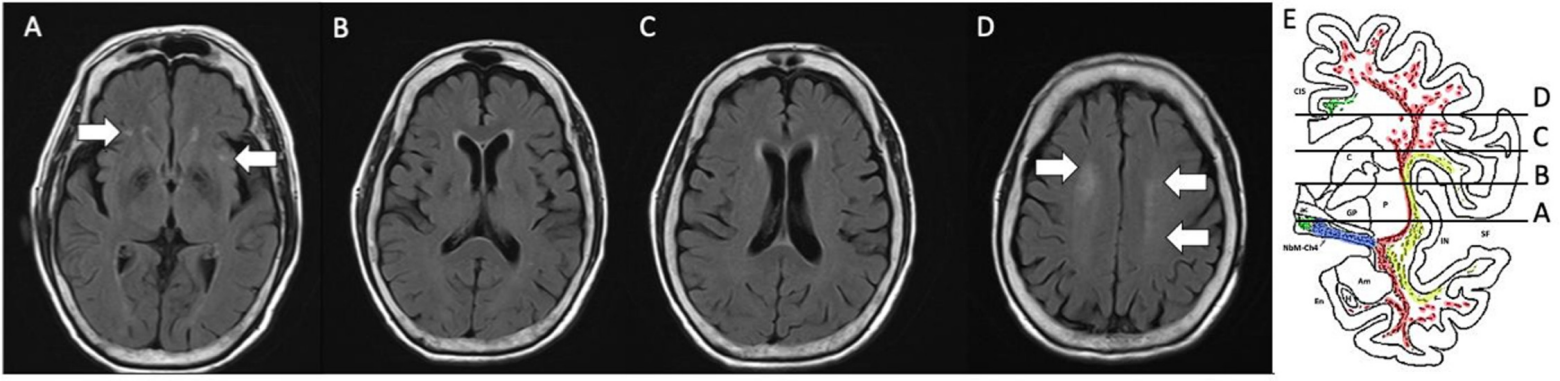
Sample of T2-weighted-FLAIR sequence brain MRI images of four anatomical landmarks for CHIPS score assessment. (A) Lower portion of the external capsule: anterior (right = 1, left = 1), posterior (right = 0, left = 0); add scores and multiply by a factor of 4 for a subtotal of 8 (B) Upper portion of the external capsule: anterior (right = 0, left = 0), posterior (right = 0, left = 0), cingulate (right = 0, left = 0); add scores and multiply by a factor of 3 for a subtotal of 0 (C) Corona radiata: anterior (right = 0, left = 0), posterior (right = 0, left = 0), cingulate (right = 0, left = 0); add scores and multiply by a factor of 2 for a subtotal of 0 (D) Central semiovale: anterior (right = 2, left = 1), posterior (right = 0, left = 1); add scores and multiply by a factor of 1 for a subtotal of 4. Total CHIPS score: 12 (E) Redrawing of a schematic of cholinergic pathway trajectories with permission from Mesulam MM [29]. Note: CHIPS: Cholinergic Pathways Hyperintensity Scale.

**Fig 2:**
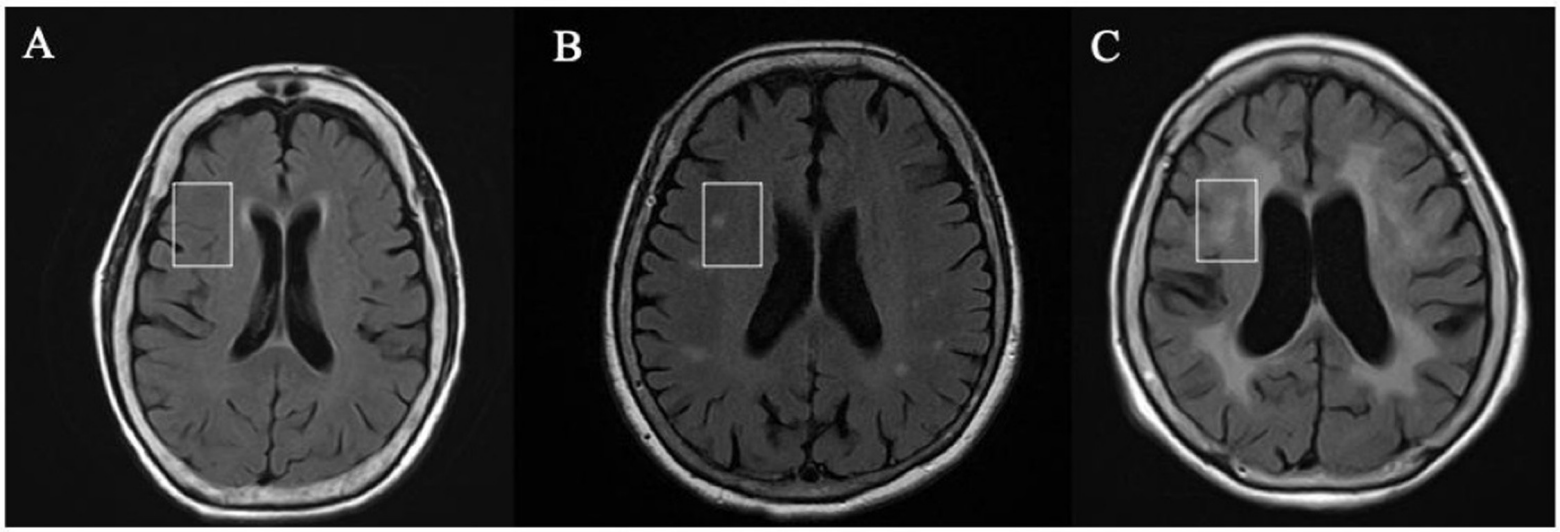
Brain MRI images of WMH severities corresponding to CHIPS scores as circumscribed by white rectangles. (A) Absence of WMHs (CHIPS score: 0) (B) Mild WMHs (CHIPS score: 1), as indicated by the WMHs occupying less than 50% of the marked area (C) Moderate-to-severe WMHs (CHIPS score: 2), as indicated by the WMHs occupying more than 50% of the marked area Note: WHM white matter hyperintensities, CHIPS: Cholinergic Pathway Hyperintensity Scale.

The CHIPS was employed to evaluate the severity of cholinergic white matter hyperintensity. It was measured in medial pathway (cingulate gyrus white matter) and lateral pathway (external capsule and claustrum) which were separated into 10 regions, using major anatomical landmarks on 4 index slices. These anatomical landmarks of 4 index slices were (A). lower external capsule, (B). upper external capsule, (C). corona radiata level, and (D). central semiovale levels respectively. Each landmark was measured bilaterally from anterior to posterior as in (Fig 1), with severity of scores ranging from 0 to 2 (0: normal, 1: mild, 2: moderate to severe (Fig 2)).

Each slice was weighted to account for the decreasing concentration of cholinergic fibers as they project up and fan out in the white matter (maximum weight (4) for lower external capsule level; minimal weight (1) for central semiovale level). The intra-class correlation coefficient was 0.94.

### Statistical analysis

All analyses were conducted using IBM SPSS Statistics for Windows, version 26 (IBM Corp., Armonk, NY, USA). The independent t test was performed for between-group comparisons of baseline demographic data (e.g., age, education level, and cognitive status) and MMSE, CDR, CDR-SB, and neuroimaging data (scores on the CHIPS, Fazekas scale, and MTA scale). Categorical variables (CDR groups, gender, presence of hypertension and diabetes) were analyzed using the chi-square test and Fisher’s exact test. Next, we performed Pearson correlation analysis to determine the effects of all intervariable correlations. Finally, we conducted logistic regression analysis with responses to donepezil treatment as dependent variable and visual ratings (CHIPS, Fazekas and MTA scales in separate models) as independent variable, adjusting for age, sex, education level and CDR-SB.

## Results

We excluded 13 out of 114 patients due to lost follow-up. Six patients did not regularly follow up at our memory clinic. They had poor compliance with taking donepezil during the study period and we could not arrange follow-up neuropsychological test for assessing the therapeutic response. Moreover, seven patients died due to unrelated medical condition such as AMI (acute myocardial infarction), stroke or COVID pandemic. No differences existed between patients lost to follow-up and the study sample (especially in CHIPS scores). Finally, 101 participants completed the study. Of the 101 participants, 64 (63.4%) had mild cognitive impairment (CDR 0.5), 26 (25.7%) had mild dementia (CDR 1), and 11 (10.9%) had moderate dementia (CDR 2), and 52 (51.5%) patients responded to donepezil treatment. After 12 months of treatment, significant decreases in MMSE, CDR, and CDR-SB scores relative to baseline were observed in the nonresponder group. By contrast, the responder group exhibited less regression than non-responder group [Table 3].

**Table 3:** Comparisons of cognitive functions before and after 12 months of donepezil treatment in participants.

	Responders		P value	Non-responders		P value
	T0	T1		T0	T1	
MMSE score, mean (SD)	19.0 (5.1)	19.8 (4.9)	0.384	19.7 (5.1)	13.0 (5.8)	0.000*
CDR score, mean (SD)	0.7 (0.4)	0.6 (0.3)	0.078	0.8 (0.5)	1.2 (1.7)	0.001*
CDR-SB score, mean (SD)	4.2 (2.8)	4.4 (2.8)	0.769	4.6 (3.5)	8.0 (4.7)	0.000*

Note: SD standard deviation, MMSE Mini-Mental State Examination; CDR Clinical Dementia Rating; CDR-SB CDR sum of boxes; T0 pretreatment; T1 posttreatment,

* *p* < 0.05

Women were the majority in both the responder and nonresponder groups (65.4% and 61.2%, respectively), and no between-group sex differences were noted. No significant differences in baseline cognitive function or other demographic variables were detected. The demographic data of both the groups are presented in Table 2 as above.

On baseline MRI images, mean CHIPS scores were significantly lower in responders (14.9 ± 9.2 vs. 21.1 ± 12.9, P = 0.007; Table 2). Neither the Fazekas nor MTA scores exhibited any between-group differences. Notably, CHIPS scores (r = 0.19, P = 0.046) and MTA scores (r = 0.27, P = 0.006) were correlated with age, but the response to donepezil and age at baseline did not show any correlations. Because vascular risk factors are highly related to cognitive impairment, we examined the links between the comorbidities of hypertension and diabetes mellitus with responses to donepezil and there was no significant correlations. The multiple logistic regression analysis revealed a significant association between CHIPS scores and responses to donepezil (b = 0.950, standard error [SE] = 0.020, P = 0.013) after adjustments for baseline age, sex, education level, and CDR-SB scores. The p-value of the model was 0.013. However, neither the Fazekas scores (b = 0.676, SE = 0.323, p = 0.227) nor MTA scores (b = 1.090, SE = 0.099, P = 0.388) was associated with responses to donepezil [Tables 4 and 5].

**Table 4:** Stepwise Logistic regression models for prediction of donepezil response.

Models		SE	(Exp)B	95% CI	p value
1	Age at baseline	0.029	0.948	0.896–1.003	0.063
2	Age at baseline	0.029	0.948	0.896–1.003	0.064
	Sex	0.421	0.847	0.371–1.933	0.694
3	Age at baseline	0.029	0.948	0.895–1.003	0.061
	Sex	0.453	0.874	0.732–2.051	0.757
	Education level	0.045	0.987	0.904–1.079	0.777
4	Age at baseline	0.031	0.938	0.884–0.996	0.077
	Sex	0.438	0.861	0.365–2.033.	0.733
	Education level	0.049	1.011	0.917–1.114	0.828
	CDR-SB score	0.066	0.983	0.863–1.120	0.800

Note: Stepwise logistic regression of prediction of responsiveness to donepezil with age at baseline, gender,

and education level as independent variables. SE standard error; CI confidence interval; CDR-SB Clinical Dementia Rating sum of boxes; MMSE Mini-Mental State Examination.

**Table 5:** Logistic regression models for prediction of donepezil response in association with CHIPS-score, Fazekas-score, and MTA-score.

Models		SE	(Exp)B	95% CI	P value
5A	Age at baseline	0.023	0.963	0.905–1.025	0.234
	Sex	0.459	0.970	0.394–2.386	0.947
	Education level	0.048	0.970	0.884–1.065	0.523
	CDR-SB score	0.071	0.942	0.820–1.082	0.400
	CHIPS score	0.020	0.950	0.913–0.989	0.013*
5B	Age at baseline	0.031	0.957	0.901–1.016	0.151
	Sex	0.443	0.835	0.350–1.992	0.685
	Education level	0.046	0.978	0.894–1.071	0.638
	CDR-SB score	0.068	0.972	0.851–1.110	0.678
	Fazekas score	0.323	0.676	0.359–1.275	0.227
5C	Age at baseline	0.031	0.941	0.885–1.001	0.054
	Sex	0.459	0.772	0.314–2.898	0.573
	Education level	0.046	0.985	0.901–1.077	0.744
	CDR-SB score	0.067	0.987	0.866–1.125	0.844
	MTA score	0.099	1.090	0.897–1.324	0.388

Note: Stepwise logistic regression of prediction of responsiveness of donepezil with age at baseline, gender, and education level as independent variables and CHIPS, Fazekas, and MTA scores as dependent variables. SE standard error; CDR-SB Clinical Dementia Rating sum of boxes; MMSE Mini-Mental State Examination,

* mean p value <0.05

## Discussion

This study clarified the relationship between the integrity of the cholinergic tract and responsiveness to AChEI. At baseline, responders had significantly lower CHIPS scores than nonresponders [Table 2]. Overall, nonresponders exhibit more significant clinical regression (based on our definition) in [Table 3]. We conducted a logistic regression analysis to predict responses to AChEI treatment by using scores on the CHIPS, Fazekas scale, and MTA scales as variables. Considering that age, education level, and baseline dementia severity may influence the rate of cognitive decline, adjustments were made for these factors. Our results suggest that the severity of WMHs in the cholinergic pathway helps predict responsiveness to AChEI treatment.

A substantial body of evidence indicates that diffuse WMHs in the brain are associated with the clinical severity of amnestic MCI and AD [38], as well as with the cognitive decline in these conditions. However, the link between cholinergic modulation and AChEI response remains to be established. Herein, the severity of WMHs in the cholinergic pathways was the only difference between the responder and nonresponder groups. Moreover, adjusting for the effect of global cognitive function (CDR-SB scores), more WMHs in the cholinergic pathways were associated with poorer responsiveness to AChEI treatment. Our results demonstrate that WMHs in the cholinergic pathway play a more decisive role in determining responsiveness to AChEI treatment than do WMHs in general. Similar observations have been presented in the literature, with one study reporting that white matter lesions in the frontal lobe and basal ganglia significantly reduced responsiveness to AChEI treatment [15]. Mounting evidence suggests links between the distribution or location of WMHs in the brain and cognition [38, 39]. One investigation noted that specific white matter tracts in the brain contributed more significantly than others to the conversion of MCI to AD and the progression of AD [40]. Another study reported by Fukui et al. also supported our primary result that higher CHIPS may hinder the efficacy of the AChEI (donepezil) [18]. However, there were also a few studies with different views. One study reported by Hongo et al. [17] stated that regional cerebral blood flow rather than CHIPS score could predict the response of the AChEI (donepezil) better. In another study conducted by Ho et al., one of their results showed that the generalized WMH was not different between responder and non-responder of the AChEI (rivastigmine) [26]. But, they also found that more WMH related to better cognitive outcomes after one year of AChEI (rivastigmine) therapy. Our study did not change the current results even using the same response criteria as Ho et al., (no deterioration on MMSE and CDR-SB). The results of previous studies were still very conflicting. Some of these studies used visual rating scales for generalized WMH and others did not apply a comprehensive score to evaluate the response of AChEI. Tracking the reduction of specific white matter tracts may be a promising direction to pursue in research on the monitoring of AD progression and responses to AChEI treatment. The specific mechanism underlying the correlation between WMHs in the brain and the progression of MCI to AD or the progression of AD has yet to be elucidated. Several hypotheses have been advanced. Most studies predominantly regarded WMHs as a presentation of small vessel disease [41]. However, WMH is sometimes considered a vascular form of amyloid deposition, not necessarily associated with vascular risk factors such as hypertension or stroke. In one study, the severity and topographic pattern of WMHs was correlated with amyloid load and amyloid distribution in the brain [42]. Still, WMHs could be driven by other pathological processes of neurodegeneration. In one recent study evaluating the cholinergic integrity in cognitively unimpaired individuals, WMH load was not correlated with amyloid or tau amount [43].

Herein, the mean age of the nonresponders was slightly higher (79.3 ± 7.1 years vs. 76.6 ± 7.4 years). Nonresponder groups may exhibit more neurodegenerative pathological changes than responder groups. Cohort studies employing neuroimaging modalities such as amyloid PET to examine cerebral microbleeds as a marker of diffuse vascular and neurodegenerative brain damage may provide further insight into the pathophysiological implications of WMHs. Some earlier studies predicted responses to AChEI treatment by considering hippocampal size [13, 14]. In one investigation, poor response was associated with younger age at AD onset and more severe hippocampal atrophy, but WMHs did not contribute to the prediction of this response [14]. Notably, because the median follow-up duration in that study was 46.6 months, hippocampal atrophy possibly reflected the clinical progression of AD rather than poor responses to AChEI treatment. Given that hippocampal volume has been established as a meaningful predictor of MCI conversion and cognitive decline [14]. However, evidence supporting its role in predicting responses to AChEI is inconsistent as described in Table 1. Most of our participants had mild cognitive impairment to mild dementia (CDR score: 0.5 to 1); in other words, these patients were in a relatively early stage of the clinical course. Therefore, we believe that the integrity of the cholinergic system (particularly the cholinergic pathway) was the primary determinant of responsiveness to AChEI treatment in this group.

This study has some limitations. First, the absence of a control group without AChEI treatment is an important limitation of the study. Since the responders showed lower CHIPS at baseline, part of the effect may be explained by brain status at baseline. It seems that the integrity of the cholinergic pathway can be one of the important factors of brain status. Second, changes in cognitive function were only evaluated through MMSE and CDR scores. However, responses to AChEI treatment may differ between specific cognitive domains. Prospective studies can compare the difference between specific cognitive domains before and after AChEI treatment. Third, we did not have APOE genotyping status of all participants and could not evaluate the relation between the presence of APOE4 carrier status and AChEI response. APOE4 also related to the exacerbation of white matter hyperintensities and cognitive deterioration in AD. However, many evidence clarified that APOE4 status does not significantly influence the response of AChEI [6]. Fourth, we examined cholinergic pathways, general WMHs, and hippocampal atrophy by using visual rating scale. Future studies can employ neuroimaging techniques such as diffusion tensor imaging or automated volumetry. Fifth, AD biomarkers were not applied in this study. The diagnosis of AD was based on longitudinal follow-up (at least 12 months) and clinical criteria.

### Conclusion

Our results suggest that WMHs in the cholinergic pathway, not diffuse white matter lesions or hippocampal atrophy, are correlated with poorer responsiveness to AChEI treatment. Therefore, further explorations into the role of the cholinergic pathway in AD are imperative.

## Supporting information

S1 File Minimal data set for each participant. (PDF)
