# Supplementary material for: White matter hyperintensities in cholinergic pathways may predict poorer responsiveness to acetylcholinesterase inhibitor treatment for Alzheimer’s disease
Source: PLoS One. 2023 Mar 31;18(3):e0283790. doi: 10.1371/journal.pone.0283790 (PMC10065432; doi:10.1371/journal.pone.0283790)
Supplement: S1 File — (PDF) [file pone.0283790.s001.pdf]

Patient ID	Response or not	Gender	Hypertension	Diabetes mellitus	Age	Education level
1	response	male	nil	present	79	3
2	response	female	nil	nil	84	0
3	response	female	present	present	82	6
4	response	male	present	nil	76	18
5	response	female	present	present	69	12
6	response	female	nil	nil	85	5
7	response	male	present	present	84	6
8	response	male	nil	nil	76	9
9	response	female	nil	nil	80	12
10	response	female	present	present	80	0
11	response	male	present	present	85	9
12	response	male	nil	nil	84	6
13	response	female	present	present	69	6
14	response	female	present	nil	88	2
15	response	female	present	nil	80	12
16	response	male	present	present	76	6
17	response	male	nil	nil	81	12
18	response	male	present	nil	85	9
19	response	female	present	present	56	6
20	response	male	present	nil	74	9
21	response	male	nil	nil	67	15
22	response	female	nil	nil	63	15
23	response	male	nil	nil	80	6
24	response	female	present	nil	60	6
25	response	male	present	present	78	16
26	response	female	present	present	74	6
27	response	female	present	present	82	12
28	response	female	present	present	79	6
29	response	female	present	present	80	0
30	response	female	nil	nil	66	6
31	response	female	nil	nil	80	10
32	response	female	present	present	69	6
33	response	male	present	nil	74	0
34	response	female	present	nil	74	3
35	response	male	present	present	73	12
36	response	female	nil	nil	73	6
37	response	female	present	nil	88	12
38	response	male	nil	nil	82	6
39	response	female	present	nil	76	6
40	response	female	nil	nil	74	6
41	response	female	present	nil	80	9
42	response	female	present	present	79	9
43	response	male	present	present	81	16
44	response	female	present	present	85	6
45	response	female	present	nil	76	9
46	response	female	present	nil	70	6

47	response	female	nil	nil	70	12
48	response	female	present	present	76	0
49	response	female	present	present	79	5
50	response	male	present	nil	59	14
51	response	female	present	nil	89	9
52	response	female	nil	nil	75	6
53	non-response	male	present	nil	66	9
54	non-response	female	nil	nil	81	9
55	non-response	female	nil	nil	82	0
56	non-response	female	present	nil	87	6
57	non-response	female	present	nil	82	9
58	non-response	female	nil	nil	83	9
59	non-response	male	present	nil	81	16
60	non-response	male	present	nil	76	9
61	non-response	female	present	nil	88	6
62	non-response	male	nil	nil	92	16
63	non-response	female	present	present	83	12
64	non-response	female	present	present	78	0
65	non-response	female	present	present	78	5
66	non-response	male	present	nil	83	0
67	non-response	female	present	nil	72	9
68	non-response	male	nil	nil	81	0
69	non-response	female	present	present	71	6
70	non-response	female	present	present	75	12
71	non-response	female	present	present	74	6
72	non-response	female	present	present	76	0
73	non-response	female	present	nil	77	6
74	non-response	male	present	nil	73	12
75	non-response	male	nil	nil	64	18
76	non-response	female	present	nil	89	6
77	non-response	male	nil	nil	85	15
78	non-response	male	present	present	65	12
79	non-response	female	present	present	71	16
80	non-response	male	present	nil	71	12
81	non-response	female	present	nil	90	6
82	non-response	female	present	present	75	6
83	non-response	female	present	present	76	0
84	non-response	male	nil	nil	81	9
85	non-response	male	present	nil	82	1
86	non-response	male	nil	nil	85	0
87	non-response	female	present	present	90	6
88	non-response	male	nil	nil	91	6
89	non-response	female	present	present	71	12
90	non-response	female	nil	nil	84	16
91	non-response	female	nil	nil	79	2
92	non-response	female	present	present	71	6
93	non-response	female	present	present	89	6
94	non-response	female	nil	nil	89	12

95	non-response	male	nil	nil	74	6
96	non-response	female	nil	nil	76	6
97	non-response	male	present	nil	90	15
98	non-response	male	present	nil	80	6
99	non-response	female	present	nil	80	6
100	non-response	male	nil	nil	79	14
101	non-response	female	present	present	74	9

Patient ID	CHIPS	Fazekas	MTA	MMSE	CDR	CDR-SB
1	17	2	4	20	0.5	3.5
2	23	2	2	9	2	9
3	28	2	4	20	1	4
4	19	1	0	23	0.5	2.5
5	4	1	0	20	0.5	5
6	18	2	0	6	2	12
7	24	1	3	12	2	12
8	25	1	8	14	1	6
9	19	1	0	20	0.5	4.5
10	3	1	0	11	0.5	4
11	25	2	0	18	0.5	4.5
12	1	0	7	21	0.5	1
13	18	1	0	18	0.5	4.5
14	0	0	3	22	1	4
15	21	2	1	29	0.5	0.5
16	23	2	0	20	0.5	1
17	23	2	8	27	0.5	1
18	7	1	2	19	0.5	3.5
19	4	1	0	22	0.5	3
20	18	1	3	28	0.5	1
21	11	1	3	20	0.5	3.5
22	15	1	0	22	0.5	4
23	0	0	4	19	0.5	3.5
24	4	1	0	16	0.5	4.5
25	15	1	8	18	0.5	3
26	10	1	0	15	1	7
27	21	2	7	26	0.5	2
28	28	2	2	23	0.5	2.5
29	33	2	3	10	1	7
30	5	1	0	22	0.5	1.5
31	12	1	0	17	1	9
32	8	1	0	22	1	4.5
33	22	2	0	17	0.5	2
34	18	1	0	19	1	4.5
35	27	2	3	22	0.5	2
36	6	1	2	26	0.5	2.5
37	33	2	2	23	1	4
38	18	2	2	16	1	5.5
39	13	1	2	17	0.5	6
40	5	1	1	23	0.5	3.5
41	4	1	0	22	0.5	2.5
42	13	1	0	26	0.5	0.5
43	24	1	8	24	0.5	2.5
44	15	1	2	14	0.5	1.5
45	0	0	0	12	1	6.5
46	0	0	4	10	1	7.5

47	15	1	0	18	0.5	3
48	16	2	3	20	0.5	3
49	28	3	5	16	1	6
50	5	1	2	27	0.5	2
51	23	3	6	15	2	12
52	12	1	1	13	1	6.5
53	9	1	0	17	1	6
54	21	2	2	16	0.5	4
55	42	3	5	9	1	4.5
56	28	2	3	18	0.5	3
57	29	2	2	21	1	5
58	38	1	3	21	0.5	1.5
59	21	1	4	26	0.5	1
60	46	2	6	23	0.5	2.5
61	15	1	2	10	2	15
62	27	2	3	11	1	8
63	21	1	0	21	1	5
64	6	1	2	15	2	14
65	10	1	0	13	0.5	4.5
66	57	3	0	14	0.5	1.5
67	41	2	0	23	0.5	1.5
68	49	3	3	18	0.5	2
69	18	1	3	23	0.5	1
70	16	1	0	23	0.5	2
71	31	2	0	27	0.5	4
72	30	2	3	20	0.5	3
73	16	2	0	28	0.5	1
74	37	2	6	24	0.5	3
75	5	1	0	19	1	9
76	6	1	0	13	2	10
77	13	1	0	19	0.5	5.5
78	24	1	3	26	0.5	3
79	17	1	3	24	0.5	3.5
80	12	1	0	26	0.5	6
81	23	2	5	16	1	6
82	25	2	3	22	0.5	3
83	21	1	2	23	0.5	3.5
84	20	1	3	19	1	4.5
85	17	1	2	16	0.5	1
86	28	1	1	21	0.5	1.5
87	20	2	4	16	0.5	2.5
88	11	2	7	8	2	11
89	9	1	1	18	2	10
90	24	2	4	22	2	11
91	4	1	4	10	2	8
92	33	2	0	24	0.5	1
93	3	1	0	22	1	7.5
94	4	1	0	27	1	3

95	2	0	4	18	0.5	4
96	25	2	2	22	0.5	0.5
97	8	1	2	25	1	4.5
98	33	1	0	16	1	10
99	22	2	4	28	0.5	0.5
100	4	1	4	23	0.5	2.5
101	17	2	0	22	0.5	3.5
